# Dyslipidemia, obesity and other cardiovascular risk factors in the adult population in Senegal

**DOI:** 10.11604/pamj.2014.19.181.4872

**Published:** 2014-10-21

**Authors:** Dominique Doupa, Sidy Mohamed Seck, Charles Abdou Dia, Fatou Agne Diallo, Modou Oumy Kane, Adama Kane, Pape Madieye Gueye, Maimouna Ndour Mbaye, Lamine Gueye, Modou Jobe

**Affiliations:** 1 Laboratoire de Biochimie-Biologie Moléculaire, Unités de Formation et de Recherche (UFR), Santé Université Gaston Berger de Saint-Louis, Sénégal; 2 Service de Néphrologie, Unités de Formation et de Recherche (UFR), Santé Université Gaston Berger de Saint-Louis, Sénégal; 3 Laboratoire de Biochimie-Biologie Moléculaire, Faculté de Médecine, de Pharmacie et d'Odontologie de l'Université Cheikh Anta Diop de Dakar, Sénégal; 4 Laboratoire de Physiologie, Faculté de Médecine, de Pharmacie et d'Odontologie de l'Université Cheikh Anta Diop de Dakar, Sénégal; 5 Service de Cardiologie, CHU Aristide Le Dantec, Dakar, Sénégal; 6 Laboratoire de Biochimie pharmaceutique, Faculté de Médecine, de Pharmacie et d'Odontologie de l'Université Cheikh Anta Diop de Dakar, Sénégal; 7 Service de Médecine Interne, Centre Hospitalier Abass Ndao, Dakar, Sénégal; 8 Laboratoire de Physiologie, Unités de Formation et de Recherche (UFR), Santé Université Gaston Berger de Saint-Louis, Sénégal

**Keywords:** Dyslipidemia, obesity, cardiovascular, risk factors, Saint Louis

## Abstract

**Introduction:**

According to the WHO, 50% of deaths worldwide (40.1% in developing countries) are due to chronic non-communicable diseases (NCDs). Of these chronic NCDs, cardiovascular diseases remain the leading cause of death and disability in developed countries. The Framingham study has shown the importance of hypercholesterolemia as a primary risk factor. In Senegal, the epidemiology of dyslipidemia and obesity are still poorly understood due to the lack of comprehensive studies on their impact on the general population. This motivated this study to look into the key epidemiologic and socio-demographic determinants of these risk factors.

**Methods:**

It was a cross-sectional descriptive epidemiological survey which included 1037 individuals selected by cluster sampling. Data were collected using a questionnaire following the WHO STEPwise approach. Socio-demographic, health and biomedical variables were collected. P value <0.05 was considered to be statistically significant.

**Results:**

The average age was 48 years with a female predominance (M: F of 0.6). The literacy rate was 65.2% and 44.7% of participants were from rural areas. The prevalence of hypercholesterolemia, hyperLDLemia, hypoHDLemia, hypertriglyceridemia and mixed hyperlipidemia were 56%, 22.5%, 12.4%, 7.11% and 1.9% respectively. One in four was obese (BMI> 30kg/m2) and 34.8% had abdominal obesity. The main factors significantly associated with dyslipidemia were obesity, urban dwelling, physical inactivity and a family history of dyslipidemia.

**Conclusion:**

The prevalence of dyslipidemia, obesity and other risk factors in the population was high needing immediate care for those affected and implementation of prevention strategies.

## Introduction

According to the WHO, Africa is the only continent in the world where communicable diseases continue to be the main cause of death. However it predicts that in the next 20 years, the main cause of death will be related primarily to ischemic heart disease, followed by cerebrovascular diseases [1]. Most of these cardiovascular diseases are the result of clinical complications resulting from arterial atheromatous lesions. Among the primary risk factors for these lesions, the Framingham study has shown dyslipidemia, particularly hypercholesterolemia to be the major triggering factor [2]. There are only a few studies on the prevalence of dyslipidemia in developing countries in general and Senegal in particular. The objectives of this study were to 1) determine the prevalence of dyslipidemia and obesity in the district of Saint-Louis in Senegal and 2) study the possible epidemiological differences between urban and rural areas.

## Methods

The study protocol was approved by the Ethics Committee of the Faculty of Medicine, Pharmacy and Dentistry of Cheikh Anta Diop University, Dakar, Senegal.

**Study type**: This was a descriptive cross-sectional epidemiological survey which was conducted from the 9th of February 2012 to the 3^1st^ of March, 2012.

**Study population**: The survey was conducted in the district of Saint-Louis in Senegal. Formerly, the capital of Senegal during the colonial era and the country's third largest city, it is located 270 km north of Dakar. It is bounded to the north by the river Senegal which separates the Republic of Senegal and the Islamic Republic of Mauritania, to the south by the Region of Louga, to the east by the district of Dagana and to the west by the Atlantic Ocean. Its surface area is 879 km2 and its population is estimated at 256,918 (2008 estimate) inhabitants. The majority of the population are women constituting 130,466 (50.8%) of the population. The population density is 292 inhabitants /km2.

**Sampling**: Individuals were selected based on a cluster sampling at two levels. Localities were first selected randomly from the list of villages and towns of the district, and then individuals were selected from each locality in proportion to the size of their population. The sample size was 1037 people, from a projection of 800 people, being a surplus of 29%, increasing the power and reliability of results.

**Selection criteria**: To be eligible for inclusion into the study, participants had to be at least 18 years of age and to have resided in the area for at least one year. These individuals were also to be available and freely accepted to participate in the study by providing a written consent according to the amendments and criteria of the Helsinki II. Individuals declining to sign the consent form, pregnant women and those who did not meet the minimum requirements for laboratory tests (e.g. fasting state) were not included.

**Data collection and study variables**: Data were collected using the WHO STEPwise approach, modified and adapted to the context of St. Louis. Data collection was carried out by 4 mobile teams each comprising investigators, including a doctor or laboratory personnel (team leader). All investigators underwent prior training for standardization or harmonization of measures to avoid discrepancies. Data were collected at the home of individuals surveyed in the mornings between 8am and 10am depending on the location. For some sites including remote villages or wards, the individuals selected for investigation were grouped at the nearest health facility.

The variables studied were: Epidemiological variables: age, sex, occupation, educational level; Health variables: history of dyslipidemia, obesity, smoking, alcohol consumption, physical inactivity; Biomedical variables measuring the height, weight, waist circumference, hip circumference, biological data mainly markers of lipid profile (total cholesterol, HDL cholesterol, LDL cholesterol, triglycerides).

### Operational definitions of variables

**The cardiovascular risk factors** considered in this study were: dyslipidemia, obesity, active tobacco smoking, alcohol consumption, physical inactivity, and a family history of dyslipidemia in first degree relatives (father, mother, brothers, sisters). The most gainful **employment or professional activity** was chosen. **Physical inactivity** was defined as the absence of daily physical activity or physical activity of less than 150 minutes per week. **Dyslipidemia** was defined by one or more of the following abnormalities: total cholesterol > 2 g /L, hypertriglyceridemia> 1.5 g /L, LDL> 1.6 g /L, HDL < 0.5 g /L in women and <0.4 g/L in men.

**The body mass index** (BMI) was calculated as the ratio of the weight (kg) to the square of height (in m). The individuals were considered: underweight if BMI ≥18 and m^2^, normal if BMI ≥18 and <25 kg/m^2^, overweight between 25 and 30 kg/m^2^ BMI, obese if BMI ≥30 kg/m^2^. **Abdominal obesity** was defined according to the National Cholesterol Education Program (NCEP) [3] by a waist circumference greater than 102 cm in men and 88 cm in women.

### Statistical analysis

Data was entered by operators in Epidata then analyzed using SPSS 16.0 software (Chicago, IL, SPSS Inc.). The results of the univariate analysis were expressed as numbers, percentages and averages together with their standard deviations. The difference was considered to be statistically significant when p <0.05. Bivariate analysis allowed us to obtain correlation coefficients to explain the relationship between quantitative variables whilst the odds ratio with its confidence interval was used to quantify the strength of the link.

## Results

**Figure 1:**
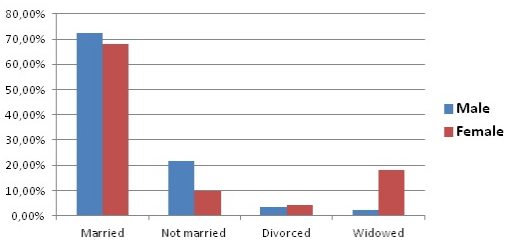
Distribution of the study population by marital status

One thousand and thirty-seven (1037) individuals were included in our study, 646 women (62% of the sample) and 391 men (38% of the sample). The sex ratio (male/female) was 0.66. We noted that 50.8% of the study population was under the age of 50 years. 70.3% of the total population was married, 15.7% single, 10.5% widowed, and 3.85% divorced. This distribution masks the disparities by gender (p <0.001). There were more unmarried individuals amongst men (21.7% versus 9.7%) and more widows among women (17.9% versus 2.4%) (Figure 1). Nearly 45% of our study population lives in rural areas and 55% in urban areas. The general characteristics of the individuals surveyed are summarized in Table 1.

**Table 1:** General characteristics of the individuals surveyed

Characteristics	Overall	Urban	Rural	p-value
Average age (years)	47.6**±**15.7	51.6**±**15.7	43.4**±**17.2	0.01
Sex ratio (M/F)				<0.01
Individuals aged (25-55ans)	0.6 50.8	0.55 55	0.8 46	0.01 <0.01
Education	65.2	73.2	16.3	<0.01
Obesity (BMI > 30kg/m^2^)	25	35	12	0.543
Abdominal obesity	34.8	45	24.6	0.035
Cholesterol (>2g/L)	56	66.4	55	0.410
LDL (>1.6g/L)	22.5	38	32	<0.01
HDL (<0.40g/L)	12.4	52	32	<0.01
Triglycerides (>1, 3g/L)	7.1	10	5	<0.01
Physical activity	39.5	55.2	24.2	<0.01
House wives	26.9	36.8	22.6	<0.01
Married	70.3	65.5	72.3	

The literacy rate in our study population was 65.2%, almost half of whom attended formal, western type of education (49%). Most of the population attained primary level of education, followed by senior secondary, then by junior secondary level of education. The dominant occupation consisted of housewives making 26.9% of the population. Active smoking was found in 4.2% of the population; being twice as much in urban compared to rural areas (5.2% versus 2.9%). On the other hand, alcohol consumption (5.1% of the population) was more common in rural compared to urban areas (8.5% versus 1.7%) (Table 2). The distribution of body mass index in the adult population by gender showed that 25% of the population was obese which was much more prevalent in women than in men (36% versus 7%) (Table 2). Obesity was also more prevalent in urban compared to rural areas (35% versus 12%). The age group 20 to 50 years had the highest prevalence rate.

**Table 2:** Distribution of risk factors according to gender

Risk factor	Number (%)	Females (%)	Males (%)	p
Obesity	25	36	7	< 0.01
Abdominal Obesity	34.8	34.6	33.1	0.543
Hypercholesterolemia	56	61.4	53.8	0.035
HyperLDLemia	22.5	28	20	0.410
HypoHDLemia	12.4	10.4	16.8	<0.01
Hypertriglycideremia	7	9	6	<0.01
Active Tobacco smoking	4.05	0.6	9.8	0.01
Alcoholism	5.10	1.2	9.7	<0.01
Physical inactivity	39.5	49.4	29.4	<0.01
History of dyslipidemia	18.70	33.10	11.3	<0.01

More than half of our study population had regular physical exercise (60.5%), found more in the male population compared to females (70.6% versus 50.6%). Among those who performed physical exercise, only 50% had it on a regular basis and 34% had intense physical activity.

Family history of dyslipidemia was found in 18.7% of the population with 24.3% claimed ignorance of their status. The prevalence of dyslipidemia of the total population was 63.8%. Isolated hypercholesterolemia was the abnormality most commonly found i.e. in 56% of the population and was found to be more common amongst women (61.4%) compared to men (53.8%) (Table 2). It was also higher in urban compared to rural areas, 66.4% versus to 55% (Table 1). HDL cholesterol level was low in 12.4% of individuals. The decrease was more marked in women than men, 16.8% and 10.4% respectively and was higher in rural compared to urban areas (52% versus 32%). The hyperLDLemia was found in 22.5% of the population with a much higher prevalence in women than men (28% against 20%) (Table 2). This increase was greater in urban compared to rural areas (38% versus 32%). The prevalence of hypertriglyceridemia was 7.11% with a higher prevalence among women than among men (9% versus 6%), and being more marked in the urban population compared to the rural population (10% versus 5%). Mixed dyslipidemia was only present in 1.97% of the population.

Associations of cardiovascular risk factors were frequent. Obesity was significantly associated with age (p <0.001), gender (p <0.001), geographic location (p <0.001), physical inactivity (p <0.001) and family history of dyslipidemia (p <0.001) (Table 3). Hypercholesterolemia also correlated significantly (p Hypercholesterolemia also correlated significantly (p <0.01) with many cardiovascular risk factors including age, gender, geographic location, weight and waist circumference (Table 4).

**Table 3:** Association of obesity (BMI) with other cardiovascular risk factors

Variables		OR	95% CI	p-value
**Age**	(18 – 40)	1		<0.0001
	(41 – 60)	3	(2.08- 4.3)	
	(61 – 87)	2	(1.26-2.87)	
**Sex**	Female	1		<0.0001
	Male	0.16	(0.102- 0.23)	
**Geographical zone**	Rural	1		<0.0001
	Urban	4.18	(3- 5.9)	
**Marital status**	Married	1		<0.0001
	Bachelor	0.17	(0.078- 0.35)	
	Divorced and widowed	1.67	(1.14 - 2.44)	
**Education**	No	1		0.008401
	Yes	0.66	(0.50- 0.89)	
**Educational level**	Primary	1		<0.0001
	Junior and senior secondary	0.85	(0.6-1.3)	
	Tertiary	0.066	(0.025- 0.14)	
**Profession**	Administrative	1		<0.0001
	Agriculture	0.55	(0.22- 1.25)	
	Business	1.17	(1.016 -2.88)	
	Student	0.023	(0.0013-0.11)	
	Housewife	2.10	(1.32- 3.43)	
	laborer	0.43	[0.192- 0.91)	
	Fishing	1.79	(0.97- 3.29)	
	Unemployed	0.86	(0.38- 1.85)	
**Alcoholism**	No	1		<0.001
	Yes	0.14	(0.023- 0.47)	
**Tobacco smoking**	No	1		<0.001
	Yes	0.09	(0.005- 0.42)	
**Physical activity (sport)**	No	1		0.001675
	Yes	0.62	(0.45- 0.83)	
**Abdominal circumference**	Normal	1		<0.0001
	Increased	28.65	(16.4-55.06)	
**Familial obesity**	No	1		<0.0001
	Yes	6.56	(4.15 -10.6)	

**Table 4:** Association of cholesterol with other cardiovascular risk factors

Variables		Odds-ratio (OR)	95% CI	p-value
**Age**	(18 – 40)	1		<0.0001
	(41 – 60)	1.97	(2.08- 4.3)	
	(61 – 87)	1.46	(1.26- 2.87)	
**Sex**	Female	1		0.0155
	Male	0.72	(0.56-0.94)	
**Geograhical zone**	Rural	1		0.0005
	Urban	1.56	(1.2- 2.01)	
**Weight**	(25 – 65)	1		0.0002
	(66 – 105)	1.69	(1.3-2.21)	
	(106 – 145)	2.13	(1.17- 4)	
**Abdominal circumference**	Normal	1		<0.0001
	Increased	1.77	(1.36- 2.30)	

## Discussion

### Socio-demographic characteristics

One thousand and thirty-seven (1037) individuals were included in this study most of whom were women. This gender imbalance may be due to the difference in professional activities as almost all the women were housewives and therefore could more easily find time to participate in consultations. The fact that Senegal is driven by strong internal migration especially from rural to urban areas mostly amongst men [4, 5]may also explain this unequal proportion. The results also showed a distribution of different ages in this group consisting of adults. Thus the distribution of the population by age shows that the majority belonged to the age group 25- 55 years (50% of the total population). This high percentage of less than 50 years reflects the realities of the national population [6]. Education was relatively high in our study population at 62.5% of which 49% had formal/western education. This percentage is closer to the demographic and health survey of 2005 which showed that St. Louis and Thies to be the most literate regions [6].

### Health variables

Our figures on prevalence of obesity are similar to those found in some studies in Senegal. Kane in 1990, in suburban areas, noted a prevalence of obesity of 9.58% and found obesity prevalence of 19% in business environments [7]. Ndiaye et al found (in Senegal) 22% of women to have a high BMI (25kg/m2 or more) [6]. Furthermore, in Africa, STEPS surveys reporting on the prevalence of obesity in neighboring countries, found 20.9% in Mauritania, 20.1% in Libreville, for both sexes combined [8]. In our sample, the prevalence of obesity was 25% and most notable among women compared to men (36% against 7%). The prevalence was higher in urban compared to rural areas (35% versus 12%). The age group 20 to 50 years recorded the highest rates. These data are confirmed by those found by Christelle in 2011 [9], in the population of St. Louis. She noted that women were more affected by obesity (30.8%) which was 9.8 times more than that found in men (p <0.001) [10]. Pessinaba S et al [11] in another recent study conducted at the district of Dagana (Saint Louis region) found a prevalence of 23% and this rate was higher in the respective age groups of 44-54 and 55-64 years.

This relatively high prevalence in our series is related to the adoption of western lifestyle by the population. These urban populations experiencing a nutritional transition characterized by a change in diet (rich in saturated fatty acids diets, low in polyunsaturated fatty acids and fiber) over traditional ones [12]. Their lifestyle is characterized by a decreased physical activity and increased sedentary behavior which is confirmed in our series where the risk of obesity is 0.62 lower in athletes compared to non-athletes p <0.001) (Table 3). The risk of obesity was also found to be very high in individuals with a family history of dyslipidemia, being 6.58 times higher than individuals compared to those who did not (p <0.01) (Table 3). The risk of obesity was also found to be very high in individuals with a family history of dyslipidemia, being 6.58 times higher than individuals compared to those who did not (p Table 3).

In our series 4.5% of the population was smokers. This is similar to that found by Pessinaba S et al [11] which showed a rate of 5.8%. A more detailed analysis shows that active tobacco smoking was higher in urban than in rural areas (5.2% against 2.9%). If the deleterious effects of tobacco have been reported by many authors as an important factor of carotid atherosclerosis, we have noted, however, a negative correlation between the use of tobacco and the risk of obesity.

As is the case with smoking, alcohol has deleterious effects on health. In some countries, alcohol has become a habit and is consumed as a food appetizer. For others, it is used as an anxiolytic or as an antidepressant. In our study, 5.1% of the sample consumed alcohol. However, the rate of alcohol intake is higher in rural than in urban areas (8.5% versus 1.7%). This high alcohol consumption rate in rural areas is also confirmed by Diouf et al [13] who found a prevalence of 11% in the population of the Ferlo. However, we observed a decrease in the risk of obesity in alcohol consumers compared to nondrinkers (odds ratio 0.62 with p < 0.01) (Table 3). This may be related to findings by Christian Lucas [14] who concluded that the low-dose alcohol consumption has a protective effect on the heart and the vascular risk increased exponentially with the doses.

### Biomedical variables

Prevalence rates for dyslipidemia were higher than those found in most African studies and particularly those conducted in Senegal. In Mauritania and in Algeria, a prevalence of 14.3% and 14.8% respectively was found [15]. However, this prevalence is similar to that found in Ivory Coast by Lokrou et al in 1998 in diabetic patients (47.4%)[16]. Isolated hypercholesterolemia was the most common dyslipidemia encountered in our study. Two other studies have also found similar results including that of Lokrou et al in 1998 and by Thiahou et al in 2010 [16, 17]. The latter authors reported that the prevalence of isolated hypercholesterolemia was 31.19% followed by HypoHDLemia of 23.85%, mixed dyslipidemia of 21.08%, isolated hypertriglyceridemia of 16.51% and finally hyperLDLemia of 7.34%. Hypercholesterolemia in our study correlated significantly (p <0.01) with many cardiovascular risk factors including age, sex, geographic location, weight and waist circumference. Similar results were found by many authors including Pessinaba et al [11] who found a higher prevalence in women than in men being 72.3% and 47.4% [11] who found a higher prevalence in women than in men being 72.3% and 47.4%.

## Conclusion

We found in this study a high prevalence of dyslipidemia, obesity and other risk factors in the population of St. Louis. Given the role that dyslipidemia contribute in the pathogenesis of cardiovascular disease (atherosclerosis) and their impact on mortality and morbidity, urgent measures are needed for adequate management of cases but also for the implementation preventative strategies through information, education and communication programs.

## References

[1] WHO: World Health report 2008 (2008). The Global Burden of Disease.

[2] Anderson KM, Castelli WP, Levy D (1987). Cholestrol and mortality: 30 years of follow-up from the Framingham study. JAMA..

[3] National Cholesterol Education Program (NCEP) Expert Panel on Detection, Evaluation, and Treatment of High Blood Cholesterol in Adults (Adult Treatment Panel III) (2002). Third Report of the National Cholesterol Education Program (NCEP) Expert Panel on Detection, Evaluation, and Treatment of High Blood Cholesterol in Adults (Adult Treatment Panel III) final report. Circulation.

[4] Diop MC (2008). Le Sénégal des migrations: Mobilité, identités et société. Paris et Dakar, Karthala, ONU-Habitat et CREPOS.

[5] Harttgen K, Klasen S Human developpement research paper. A human development index by internal migration status.

[6] Ndiaye S, Ayad M (2006). Enquête Démographique et de Santé (EDSIV), Sénégal 2005.

[7] Kane A (1990). Contribution à l’étude épidémiologique de l'hypertension artérielle en milieu suburbain africain: cas de Pikine, Sénégal. Thèse Méd. Dakar.

[8] (2005). Enquête sur les facteurs de risque des maladies non transmissibles à Dakar(Sénégal). Selon l'approche STEPS de l'OMS, 2005.

[9] Christelle NTS (2011). Prévalence, dépistage, prise en charge et contrôle des facteurs de risque cardio-vasculaire dans la ville de Saint-Louis du Sénégal. Thèse Méd..

[10] Arroyo P, Loria A, Fernández V, Flegal KM, Kuri-Morales P, Olaiz G, Tapia-Conyer R (2000). Prevalence of pre-obesity and obesity in urban adult Mexicans in comparison with other large surveys. Obes Res..

[11] Pessinaba S, Mbaye A, Yabéta GAD, Harouna H, Sib AE, Kane Ad (2013). Enquête de prévalence des facteurs de risque cardiovasculaire en population générale à Saint-Louis (Sénégal). Ann Cardiol Angeiol (Paris)..

[12] OMS, série de rapports techniques (2003). Obésité: prévention et prise en charge de l’épidémie mondiale.

[13] Diouf M, Boetsch G, Cissé D, Tal-Dia A, Bonfil JJ (2012). Modes de vie et santé bucco-dentaire chez les populations peuls du Ferlo au Sénégal. Med Sante Trop..

[14] Lucas C (2002). AVC un problème majeur de santé publique service de neurologie et pathologie neurosaculaire hôpital Salengro-CHRU de Lille Ed médicales. Mali Medical. Septembre.

[15] MS/OMS (2006). Rapport de l'enquête sur les maladies non transmissibles selon l'approche STEPwise de l'OMS: étude de l'hypertension artérielle, du diabète et des autres facteurs de risque à Nouakchott Mauritanie.

[16] Lokrou A (1998). Hyperlipidémie et diabète en Côte d'Ivoire: étude transversale de 132 cas, Médecine d. Afrique Noire..

[17] Thiahou G, Deret K, Monde A (2010). Fréquence des bilans lipidiques et prévalence des dyslipidémies au laboratoire de Biochimie du CHU de Cocody. J sci pharm Biol..

